# Mild endothelial dysfunction in Sirt3 knockout mice fed a high-cholesterol diet: protective role of a novel C/EBP-β-dependent feedback regulation of SOD2

**DOI:** 10.1007/s00395-016-0552-7

**Published:** 2016-04-12

**Authors:** Stephan Winnik, Daniel S. Gaul, Giovanni Siciliani, Christine Lohmann, Lisa Pasterk, Natacha Calatayud, Julien Weber, Urs Eriksson, Johan Auwerx, Lambertus J. van Tits, Thomas F. Lüscher, Christian M. Matter

**Affiliations:** Department of Cardiology, University Heart Center Zurich, University Hospital Zurich, Raemistr. 100, 8091 Zurich, Switzerland; Center for Molecular Cardiology, University of Zurich, Schlieren, Switzerland; Institute of Experimental and Clinical Pharmacology, Medical University of Graz, Graz, Austria; Division of Cardiology, Department of Medicine, GZO Regional Health Center Wetzikon, Wetzikon, Switzerland; Laboratory of Integrative Systems Physiology, School of Life Science, Ecole Polytechnique Fédérale de Lausanne, Lausanne, Switzerland; Zurich Center for Integrative Human Physiology, University of Zurich, Zurich, Switzerland

**Keywords:** Sirt3, Oxidative stress, SOD2, C/EBP-β, Endothelial function

## Abstract

Sirtuin 3 (Sirt3) is an NAD^+^-dependent mitochondrial deacetylase associated with superoxide dismutase 2 (SOD2)-mediated protection from oxidative stress. We have reported accelerated weight gain and impaired metabolic flexibility in atherosclerotic *Sirt3*^−*/*−^ mice. Oxidative stress is a hallmark of endothelial dysfunction. Yet, the role of Sirt3 in this context remains unknown. Thus, we aimed to unravel the effects of endogenous Sirt3 on endothelial function and oxidative stress. Knockdown of Sirt3 in human aortic endothelial cells (HAEC) increased intracellular mitochondrial superoxide accumulation, as assessed by electron spin resonance spectroscopy and fluorescence imaging. Endothelium-dependent relaxation of aortic rings from *Sirt3*^−*/*−^ mice exposed to a normal diet did not differ from *wild*-*type* controls. However, following 12 weeks of high-cholesterol diet and increasing oxidative stress, endothelial function of *Sirt3*^−*/*−^ mice was mildly impaired compared with *wild*-*type* controls. Relaxation was restored upon enhanced superoxide scavenging using pegylated superoxide dismutase. Knockdown of *Sirt3* in cultured HAEC diminished SOD2 specific activity, which was compensated for by a CCAAT/enhancer binding protein beta (C/EBP-β)-dependent transcriptional induction of SOD2. Abrogation of this feedback regulation by simultaneous knockdown of *C/EBP*-*β* and *Sirt3* exacerbated mitochondrial superoxide accumulation and culminated into endothelial cell death upon prolonged culture. Taken together, *Sirt3* deficiency induces a mild, superoxide-dependent endothelial dysfunction in mice fed a high-cholesterol diet. In cultured endothelial cells, a novel C/EBP-β-dependent rescue mechanism maintains net SOD2 activity upon transient knockdown of *Sirt3*.

**Electronic supplementary material:**

The online version of this article (doi:10.1007/s00395-016-0552-7) contains supplementary material, which is available to authorized users.

## Introduction

Sirtuin 3 (Sirt3) is a mitochondrial deacetylase that regulates mitochondrial oxidative metabolism and detoxification from oxygen radicals [1, 3, 44, 58]. It is the third out of seven mammalian homologues of the so-called class III histone deacetylases (HDACs). They share a conserved catalytic binding motif for the oxidized form of nicotinamide adenine dinucleotide (NAD^+^), defining them as class III HDACs and concentrating their activity to times of low cellular energy levels [7, 9, 14, 19, 58]. Since its first description in 1935, caloric restriction is the only intervention that has been shown to prolong lifespan and maintain mammalian health [25, 32, 47]. Mammalian sirtuins provide protective effects in a variety of age-related pathologies, thus promoting healthy aging [7, 13, 23, 34]. Sirt3 is one out of two sirtuins that have been associated with human longevity and health span [4, 15, 46]. Cardiovascular diseases, including myocardial infarction and stroke, are the leading global cause for age-related morbidity and mortality, accounting for 17.3 million deaths a year, with an estimated rise to >23.6 million by 2030 [2, 26].

Recently, Sirt3 deficiency has been reported to accelerate the development of the metabolic syndrome, a cluster of hallmark risk factors for cardiovascular disease [18]. Although we did not observe an atherosclerotic phenotype in low-density lipoprotein receptor knockout mice lacking *Sirt3*, its loss was associated with accelerated weight gain, impaired metabolic adaptation and increased levels of systemic oxidative stress [55].

Excess endothelial reactive oxygen species (ROS), subsequent mitochondrial DNA damage and progressive respiratory chain dysfunction are essential in the development of endothelial dysfunction, an early event in and an independent predictor of cardiovascular disease [17, 28, 29, 33, 36, 40, 41, 48]. In numerous settings including embryonic development, age-related hearing loss, neuronal injury, and cardiac hypertrophy, Sirt3 has been shown to protect from oxidative stress [10, 22, 49, 51]. In the majority of settings, Sirt3 augmented superoxide scavenging by enhancing superoxide dismutase 2 (SOD2) and/or catalase (cat) activity [42, 51, 53]. Whether increased SOD2 activity is mediated by direct deacetylation or transcriptional regulation remains controversial [51, 53]. Moreover, Sirt3 activates isocitrate dehydrogenase 2 (IDH2), which drives the tricarboxylic acid (TCA) cycle and is a major donor of NADPH, an essential cofactor for both glutathione regeneration and endothelial nitric oxide synthase (eNOS)-mediated NO formation [31, 43, 44, 59]. Hitherto, only few data exist on the role of Sirt3 in *arterial* endothelial cells [35]. No data are available on its effects on endothelium-dependent vasodilation.

In contrast to the constitutive expression of the cytosolic and extracellular isoforms SOD1 and SOD3, the inducible expression of SOD2 allows the response to varying levels of intracellular oxidative stress. The acetylation-dependent transcription factor CCAAT/enhancer binding protein beta (C/EBP-β) has been shown to bind an intronic TNF-responsive element of SOD2 and thereby facilitate SOD2 gene transcription in response to various stimuli associated with increased levels of intracellular ROS [6, 21, 30].

Our goal was to unravel the effects of endothelial Sirt3 on oxidative stress and endothelial function, and to investigate the underlying mechanisms in human aortic endothelial cells. To enhance oxidative stress in aortae, we exposed mice to a high-cholesterol diet [37]. In the current work, we uncovered a C/EBP-β-dependent induction of SOD2 expression as rescue mechanism for the Sirt3-dependent loss of SOD2 activity, an interaction, that until to date remained unknown.

## Methods

### Mice

Mice were housed in a temperature-controlled facility with a 12-h light/dark cycle and free access to chow and water. All animal studies have been approved by the appropriate ethics committee and have therefore been performed in accordance with the ethical standards laid down in the 1964 Declaration of Helsinki and its later amendments. Mice with a germline *Sirt3* deletion were generated as described. [12, 55] Congenic C57BL6/J *Sirt3*^−*/*−^ mice were generated through nine generations of backcrosses with C57BL6/J mice. Eight-week-old male *Sirt3*^−*/*−^ and *wild*-*type* mice were fed a 1.25 % (w/w) cholesterol diet (research diets) for 12 weeks and subsequently killed for fasted (unless indicated otherwise) studies.

### Endothelial function

Endothelium-dependent vasorelaxation was investigated as described [37, 56]. Briefly, aortae were explanted and aortic rings were obtained. Relaxation in response to acetylcholine (ACh) or sodium nitroprusside (SNP) was assessed using isometric force transducers in organ chamber baths (Multimyograph, DMT). Maximal contraction was defined before initiating the experiment using potassium chloride (KCl) in a concentration of 80 mM. Precontraction to a maximum of 70 % maximal contraction was achieved using norepinephrine (NE) in a dose of 10^−7^ M. Dose–response curves were quantified comparing areas under the curves (AUC).

### Cell culture and transfection

Human aortic endothelial cells (HAEC, Cambrex) from passage three to eight were grown to confluence at 5 % CO_2_ and 37 °C in Endothelial Growth Medium 2 (Lonza) supplemented with 10 % fetal calf serum. Transient knockdown was performed using Lipofectamine^®^ Reagent (Life Technologies) for transfection of the following small interference RNA (siRNA): Sirt3 (5′-GCC CAA CGU CAC UCA CUA CUU TT-3′), C/EBP-β (Trilencer-27 siRNA, OriGene), SOD2 (5′-AAU GCU ACA AUA GAG CAG CUU TT-3′), scrambled (5′-UUC UCC GAA CGU GGC ACG ATT-3′), Trilencer-27 Universal Scrambled Negative Control siRNA (SR30004, Origene), and Silencer Negative Control #5 siRNA (AM4642, Ambion). Total siRNA amounts were kept equal among all experiments. Where two-stage transfections (double-knockdown of *Sirt3* and *C/EBP*-*β*) were performed, all groups in the respective experiments were transfected twice. Knockdown efficiency was assessed using expression analyses on RNA- (quantitative PCR) and protein level (western blot).

### Expression analyses

RNA isolation, reverse transcription and SYBR^®^ green-based (Applied Biosystems) quantitative PCR was carried according to standard protocols using a Quant Studio 7 Flex Real Time PCR thermocycler (Applied Biosystems) with the associated sequence detection system and software. Expression was calculated using the ΔΔ*C*_T_ method. Relative gene expression was normalized to β-actin (house-keeping gene). Western blot analyses of HAEC lysates were conducted according to standard protocols using the following specific antibodies:

anti-Sirt3 (rabbit monoclonal, Cell Signalling Technology), anti-C/EBP-β [C19] (rabbit polyclonal, Santa Cruz Biotechnology), anti-SOD2 (rabbit polyclonal, Abcam), anti-catalase (mouse monoclonal, Sigma), anti-glutathion peroxidase 1 (rabbit polyclonal, Novus Biologicals), anti-total eNOS (mouse polyclonal, BD Transduction Laboratories), anti-eNOS (pThr495) (mouse, monoclonal, BD Transduction Laboratories), anti-eNOS (pSer1177) (mouse, monoclonal, BD Transduction laboratories), anti-xanthine oxidase (rabbit polyclonal, Biorbit), anti-NADPH oxidase subunit p22^*phox*^ (rabbit polyclonal, Biorbit), and anti-β actin (mouse monoclonal, Sigma-Aldrich). Specific signals were detected using species-specific secondary antibodies.

### Immunoprecipitation

HAEC were cultured in 10 cm cell culture dishes, transfected as described above and lysed in 1 ml radioimmunoprecipitation assay (RIPA) buffer. Samples were kept on ice throughout IP steps. The lysates were pre-cleared with 30 µl washed Protein G Agarose beads (Millipore) and, after removal of the beads, incubated over night with suitable monoclonal antibodies for SOD2, C/EBP-β or Sp1, respectively. 30 µl of washed Protein G Agarose beads were added and the mixture was incubated for 4.5 h with agitation on the incubation wheel. Beads–antibody–antigen complexes were separated from the lysates by centrifugation and the pellet washed three times with RIPA buffer. After adding 30 µl of 4× Laemmli buffer, the samples were incubated at 60 °C with shaking for 10 min and the supernatant resulting from subsequent centrifugation was analyzed by western blotting.

The following antibodies were used for immunoprecipitation and subsequent determination of the acetylation or nitrosylation status, respectively: anti-SOD2 [1E8] (mouse monoclonal, Abnova), anti-acetyl lysine (rabbit polyclonal, Chemicon), and anti-nitro tyrosine [HM.11] (mouse monoclonal, Abcam).

### Electron spin resonance spectroscopy

Intracellular superoxide in HAEC was detected by electron spin resonance (ESR) spectroscopy using the superoxide-specific spin trap 1-hydroxy-3-methoxy-2,2,5,5-tetra-methylpyorrolidine (CMH, Noxygen) as described [50, 57].

### Mitochondrial superoxide detection

Mitochondrial superoxide generation was investigated based on the oxidation and fluorogenic nucleic acid binding of a mitochondrial- and superoxide-specific probe (MitoSOX™, Invitrogen). Cells were stained according to the manufacturer’s protocol and fixed with 4 % paraformaldehyde afterwards. Fluorescence was quantified using an Olympus BX51 microscope. Micrographs were quantified using ImageJ (NIH).

### Scavenging of mitochondrial superoxide

HAEC were handled and transfected as described above. Upon transfection with siRNA the medium was supplemented with 1 µM mitoTEMPO (Sigma). Medium was replaced with fresh EGM-2 medium containing 1 µM mitoTEMPO once, before harvesting the lysates for expression analyses or staining the cells for fluorescence imaging.

### Superoxide dismutase 2 (SOD2) activity

Mitochondrial fractions of HAEC were separated from whole cell lysates by centrifugation. Enzymatic activity of SOD2 in HAEC was assessed based on its capacity to dismutate superoxide radicals generated by xanthine oxidase under controlled conditions, using the Superoxide Dismutase Assay Kit (Cayman Chemical). Superoxide radicals were detected by colorimetric oxidation of tetrazolium salt to formazan dye. Any remaining activity of SOD1 and 3 was inhibited using potassium cyanide (1 mM) according the manufacturer’s instructions. Enzymatic activity was normalized to SOD2 protein expression.

### Nitric oxide production

HAEC were seeded into a 96 well plate and transfected as described above. At time of the assay, cells were incubated with Krebs buffer containing 0.25 µM 4,5-diaminofluorescein diacetate (DAF-2, Sigma) in presence or absence of 10 µM of the unspecific nitric oxide synthase (NOS) inhibitor l-N^5^-(1-Iminoethyl)ornithine hydrochloride (l-NIO, Sigma), or 0.25 µM of the DAF-2 negative control 4-aminofluorescein diacetate (4-AF-DA, Merck Millipore), respectively, for 20 min at 37 °C. Then l-arginine with or without calcium-ionophore (positive control) was added to the wells and fluorescence read at 490/525 nm (excitation/emission) to set the baseline. After 30 min the fluorescence was measured again and the percentage of nitric oxide increase calculated.

### Statistics

Metric variables were assessed for distribution using Kolmogorov–Smirnov tests. For *n* < 4 non-parametric distribution was assumed. Different groups were compared using unpaired Student’s *t*, Mann–Whitney, one-way ANOVA tests with Bonferroni multiple comparison post hoc tests or Kruskal–Wallis tests with Dunn’s post hoc analyses, where applicable. *p* values are two-sided. Significance was accepted for an alpha-error <0.05. Data are presented as mean ± SEM, if not indicated otherwise. Statistical analyses were performed using GraphPad Prism 5 for Mac OS X (GraphPad Software).

## Results

### Transient knockdown of Sirt3 increases endothelial superoxide levels

**Fig. 1:**
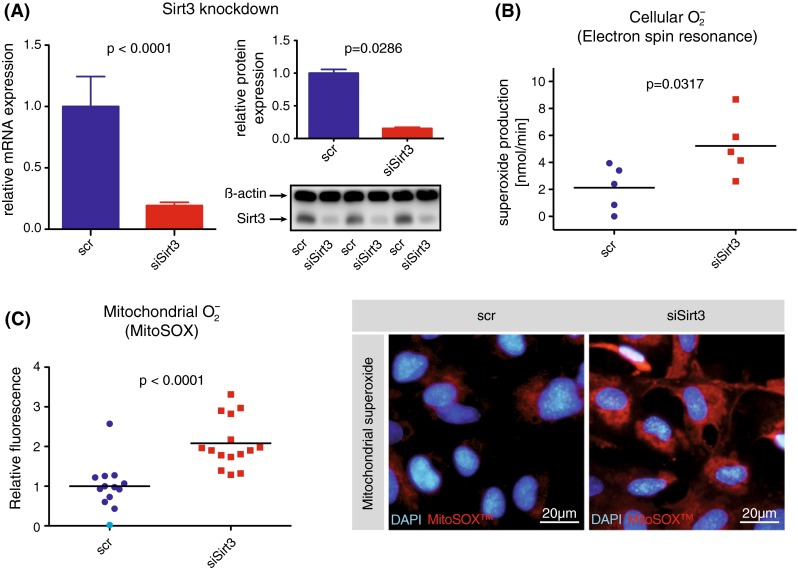
Transient knockdown of Sirt3 increases endothelial superoxide levels. **a** Quantitative PCR of mRNA (*left*) and western blot analyses of protein (*right*) isolated from human aortic endothelial cells (HAEC) following siRNA-mediated knockdown of Sirt3. **b** Electron spin resonance (ESR) spectroscopy of live HAEC following siRNA-mediated knockdown of Sirt3 to quantify intracellular superoxide release. **c** Fluorescence imaging of HAEC following siRNA-mediated knockdown of Sirt3 and detection of mitochondrial superoxide (*red*) using MitoSOX™, a mitochondrial- and superoxide-specific probe; quantification on a per cell basis; representative micrographs show nuclei (*blue*) and mitochondrial superoxide (red, MitoSOX™); *scale bars* 20 μm. At least three independent experiments, each in biological triplicates, *scr* scrambled control, *DAPI* 4′-6-diamidin-2-phenylindol, **b** and **c** show medians and single data points

siRNA-mediated transient knockdown of Sirt3 in human aortic endothelial cells (HAEC) reached an efficiency of 81 % on RNA- and 87 % on protein level (Fig. 1a). Intracellular superoxide levels were increased two-fold upon knockdown of Sirt3, as quantified using electron spin resonance spectroscopy (Fig. 1b). With Sirt3 being a mitochondrial deacetylase we used a mitochondrial- and superoxide-specific probe (MitoSOX™) to identify the cellular compartment of increased superoxide. Fluorescence imaging revealed a two-fold increase in mitochondrial superoxide levels, identifying mitochondria as the source of increased oxidative stress during transient knockdown of Sirt3 (Fig. 1c).

### Loss of Sirt3 is associated with a mild superoxide-dependent impairment of endothelial function

**Fig. 2:**
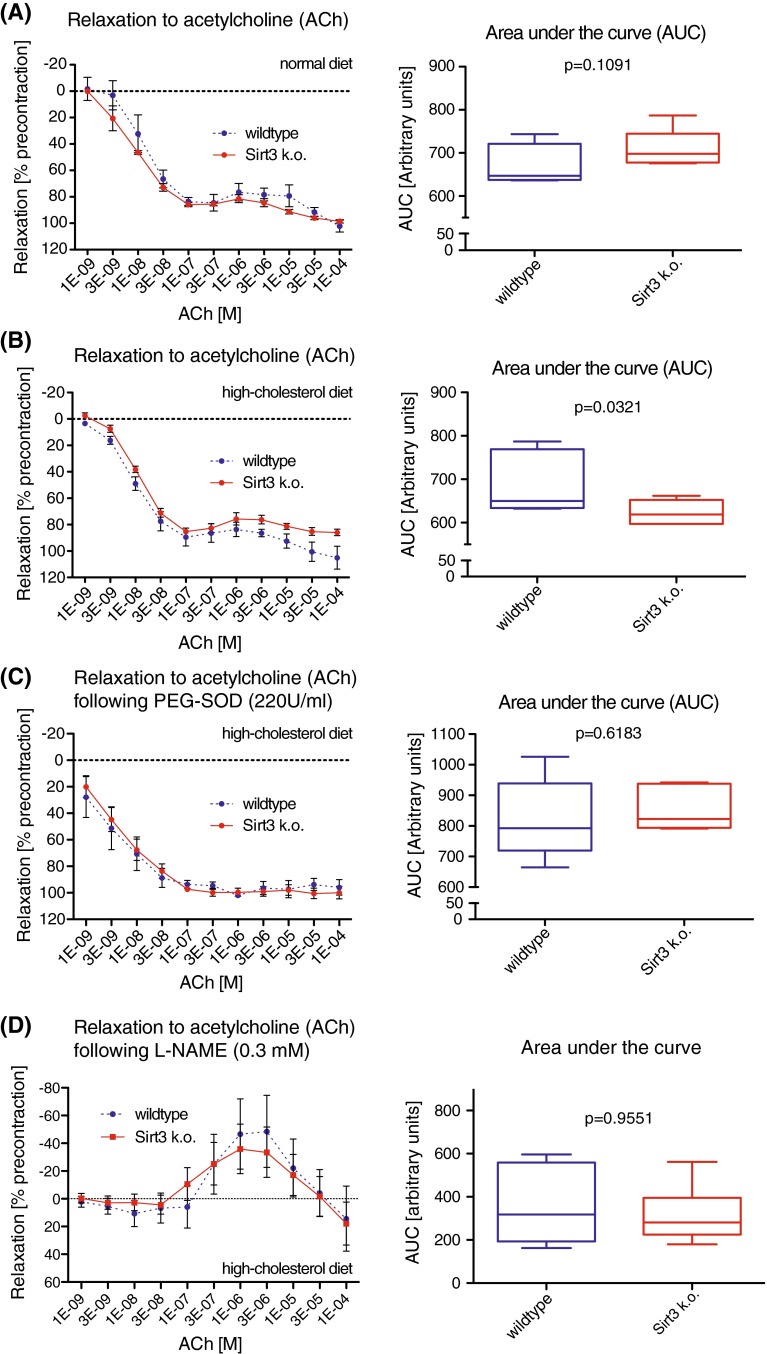
Endothelial function is mildly impaired in Sirt3^−/−^ mice in a superoxide-dependent manner. **a** Relaxation of aortic rings in response to increasing doses of acetylcholine (ACh) in *Sirt3* ^−*/*−^ compared with *wild*-*type* mice fed a regular chow. **b** As in **a** following 12 weeks of a high-cholesterol diet. **c** As in **b**, in presence of an excess of pegylated superoxide dismutase (PEG-SOD, 220 U/ml), scavenging superoxide. **d** As in **b**, following preincubation with l-NAME (0.3 mM, 30 min). *n* = 5–8 mice per group, 4–7 rings per mouse, quantification of the areas under the curve (AUC), *boxplots* show interquartile ranges, *whiskers* indicate minima and maxima

To assess the functional relevance of elevated endothelial superoxide levels in the absence of Sirt3, aortic rings of *Sirt3*^−*/*−^ and *wild*-*type* mice were explanted and endothelium-dependent relaxation was quantified in organ chamber baths. Surprisingly, aortic relaxation of *Sirt3*^−*/*−^ mice in response to acetylcholine (ACh) was unaltered compared with *wild*-*type* controls (Fig. 2a). However, upon 12 weeks of high-cholesterol diet, known to increase oxidative stress [33], aortic relaxation of both genotypes was less sensitive to ACh at low dosages and showed an overall mild impairment in aortae of *Sirt3*^−*/*−^ mice compared to *wild*-*type* controls (Fig. 2b). Scavenging endogenous superoxide by an excess of exogenous pegylated superoxide dismutase (PEG-SOD) improved the sensitivity to ACh of either genotype and abolished the impairment of aortic relaxation of high-cholesterol diet-fed *Sirt3*^−*/*−^ mice compared to *wild*-*type* controls (Fig. 2c). ACh-induced aortic relaxation in both genotypes could be prevented by preincubation with the endothelial nitric oxide synthase (eNOS) inhibitor l-nitroarginine methyl ester (l-NAME), indicating endothelial NO-dependency (Fig. 2d, S1C). Concomitantly, complete relaxation of aortae of both genotypes in response to the exogenous NO donor sodium nitroprusside (SNP) further underlined endothelium-derived NO-dependency (Fig S1A, B). Of note, there was no significant difference in body weight between *wild-type* and *Sirt3*^−*/*−^ mice (Fig S2). These findings suggest a mild, superoxide-dependent decline in aortic relaxation in the absence of Sirt3 upon a high-cholesterol diet.

### Endothelial SOD2-specific activity is diminished whereas SOD2 expression is increased following transient knockdown of Sirt3

**Fig. 3:**
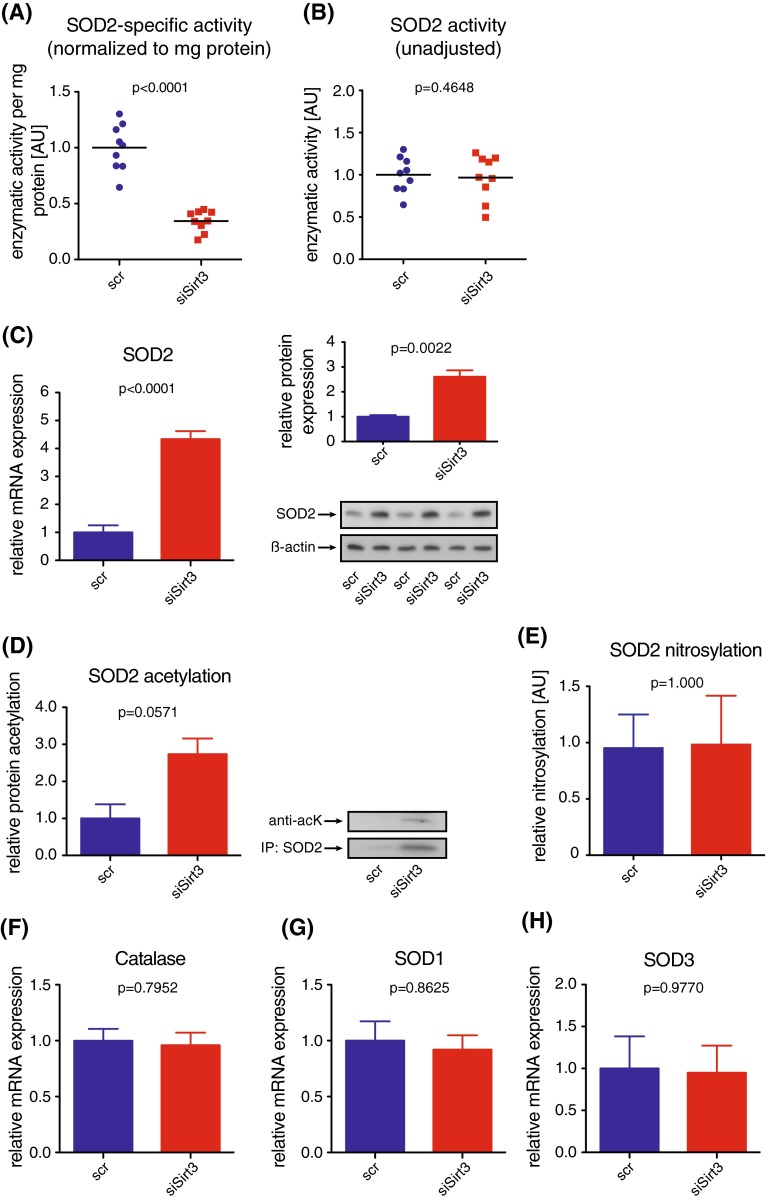
Loss of Sirt3 decreases endothelial SOD2 activity and increases SOD2 expression without affecting other ROS-scavenging or generating systems. **a** Superoxide dismutase 2 (SOD2) activity based on superoxide dismutating capacity in HAEC following siRNA-mediated knockdown of Sirt3; enzymatic activity was normalized to SOD2 protein expression; medians and single data points are shown. **b** Overall SOD2 activity, as in **a** without normalizing to protein content. **c** SOD2 mRNA (*left*) and protein (*right*) expression in HAEC following siRNA-mediated knockdown of Sirt3 using quantitative PCR and western blot, respectively. **d** Acetylation of SOD2, precipitated from HAEC following transient knockdown of Sirt3 using western blot analysis. **e** Nitrosylation of SOD2, precipitated from HAEC following transient knockdown of Sirt3 using western blot analysis. **f**–**h** Expression analyses of **f** catalase, **g** SOD1, **h** SOD3, using quantitative PCR; beta actin served as loading control in western blots, representative blots are shown. At least three independent experiments in biological triplicates were performed, *scr* scrambled control

To unravel the mechanism underlying increased endothelial mitochondrial superoxide levels upon Sirt3 deficiency, we addressed SOD2-specific activity. Following transient knockdown of Sirt3 in HAEC, superoxide scavenging capacity of SOD2 was reduced by threefold compared with controls (Fig. 3a). Unexpectedly, expression levels of SOD2 were increased by 4.4 fold on RNA- and by 2.8 fold on protein level, respectively (Fig. 3c), thus compensating for the Sirt3-dependent loss of SOD2 specific activity; overall endothelial SOD2 activity, without normalizing to protein levels, was unchanged upon transient knockdown of *Sirt3* (Fig. 3b). Decreased SOD2-specific activity was associated with a trend towards SOD2 hyperacetylation (Fig. 3d). SOD2 nitrosylation was unchanged upon knockdown of Sirt3 (Fig. 3e), suggesting a Sirt3-dependent, deacetylation-mediated activation of endothelial SOD2 under physiological conditions. Expression levels of other superoxide scavenging or decomposing enzymes, including SOD1, SOD3, catalase, thioredoxin 1, thioredoxin 2, thioredoxin-dependent peroxide reductase (PRDX3), and glutathione peroxidase were unaltered (Fig. 3f–h, Fig S3A–E). Accordingly, the expression level of endothelial superoxide-generating enzyme NADPH oxidase was unaffected by transient knockdown of Sirt3 (Fig S3F, G). Whereas no difference occurred in the cytosolic subunit p47^*phox*^ (Fig S3F), we observed a slight increase in the mRNA level of the membrane-bound subunit p22^*phox*^, which did not translate into an increased protein level (Fig S3G).

### Nitric oxide generation is not affected by Sirt3 deficiency

Assessment of endothelial nitric oxide synthase (eNOS) uncovered unaltered overall expression levels (Fig S4A) and unchanged phosphorylation at Ser1177 (Fig S4B) upon Sirt3 knockdown. However, we observed a decreased phosphorylation of Thr495 following transient knockdown of Sirt3 (Fig S4C), equivalent to an increased enzymatic activity. Together with an increased coupling of eNOS monomers (Fig S4D) this may be a compensatory effect secondary to an increased mitochondrial ROS accumulation upon Sirt3 deficiency. To assess the functional relevance of increased eNOS activity, nitric oxide (NO) generation was assessed in presence or absence of l-NIO, an unselective nitric oxide synthase inhibitor, using DAF-2 diacetate. l-NIO successfully reduced NO generation, however, no significant difference was observed upon transient knockdown of Sirt3 compared with controls, neither in presence nor in absence of l-NIO (Fig S4E). Thus, increased eNOS activity upon Sirt3 deficiency does not generate a detectable rise in NO. Nonetheless, increased eNOS coupling may contribute to counteract increased ROS levels upon Sirt3 deficiency.

### Transcriptional induction of SOD2 upon transient knockdown of Sirt3 is C/EBP-β-dependent

**Fig. 4:**
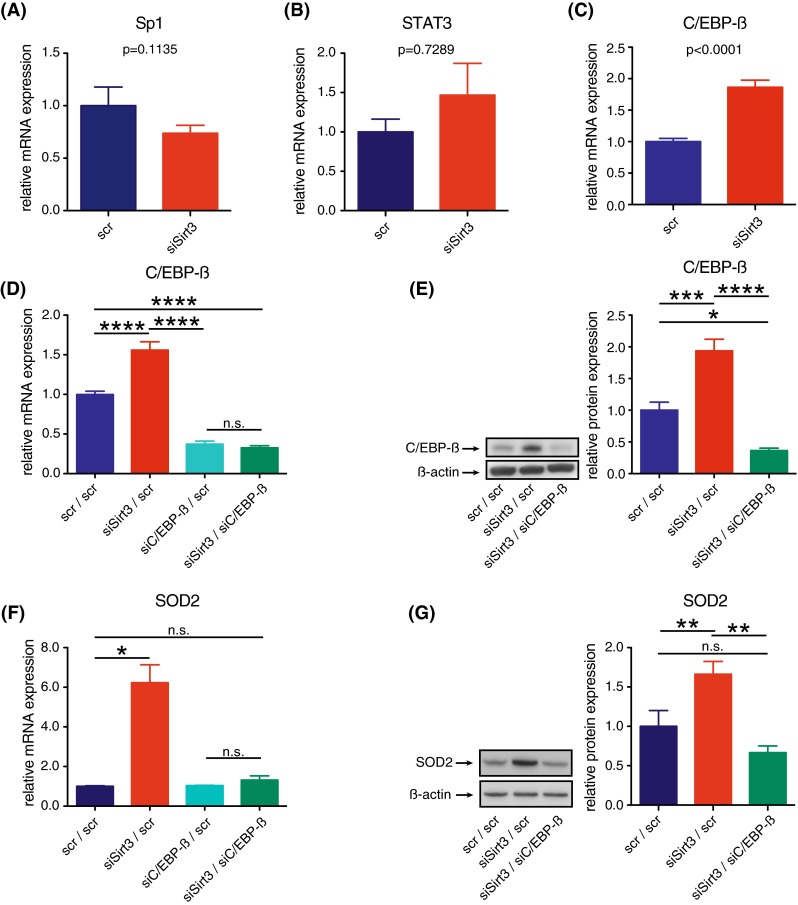
Transcriptional induction of SOD2 upon transient knockdown of Sirt3 is C/EBP-β-dependent. **a**–**d** Expression analyses of the transcription factors **a** Sp1, **b** STAT3, **c**, **d** C/EBP-β using quantitative PCR in HAEC following siRNA-mediated knockdown of Sirt3, and **d** single or simultaneous transient knockdown of C/EBP-β and Sirt3, respectively. **e** Western blot analysis of C/EBP-β in HAEC following single and simultaneous transient knockdown of C/EBP-β and Sirt3, respectively. **f**, **g** Expression analyses of SOD2 in HAEC following single or simultaneous transient knockdown of C/EBP-β and Sirt3, respectively, using **f** quantitative PCR and **g** western blot analysis. At least three independent experiments in biological triplicates were performed, *scr* scrambled control, **d**–**g** *p* values indicate overall significance by Kruskal–Wallis (**d**–**f**) or one-way ANOVA tests (**g**), differences between single groups by Dunn’s (**d**–**f**) or Bonferroni (**g**) post hoc tests are indicated separately: *n.s*. non-significant, *p < 0.05, **p < 0.01

To shed light on the unexpected transcriptional upregulation of endothelial SOD2 during transient knockdown of Sirt3, we assessed the expression levels of known, acetylation-dependent transcription factors of SOD2. Expression levels of Sp1 and STAT3 were unaffected by transient knockdown of Sirt3 (Fig. 4a, b), whereas expression of CCAAT/enhancer binding protein beta (C/EBP-β) was increased (Fig. 4c).

This differential expression in response to decreased Sirt3 levels prompted us to address the relevance of C/EBP-β in the transcriptional induction of SOD2 during transient knockdown of Sirt3. Interestingly, simultaneous knockdown of Sirt3 and C/EBP-β abrogated the transcriptional upregulation of SOD2, whereas single knockdown of C/EBP-β had no effect on SOD2 expression levels compared with controls (Fig. 4d–g).

### Transcriptional induction of C/EBP-β is SOD2-dependent

To assess the role of SOD2 on the transcriptional induction of C/EBP-β we used a loss-of-function approach in HAEC. Transient knockdown of SOD2 was associated with a significant increase in C/EBP-β transcription on RNA-level (Fig S5A), which translated into a trend towards increased protein levels of C/EBP-β (Fig S5B), indicating the existence of a direct feedback loop between SOD2 and its transcription factor C/EBP-β in endothelial cells. Sirt3 expression was unaltered (Fig S5 F, G).

### Scavenging mitochondrial superoxide does not affect Sirt3-dependent transcriptional induction of SOD2

**Fig. 5:**
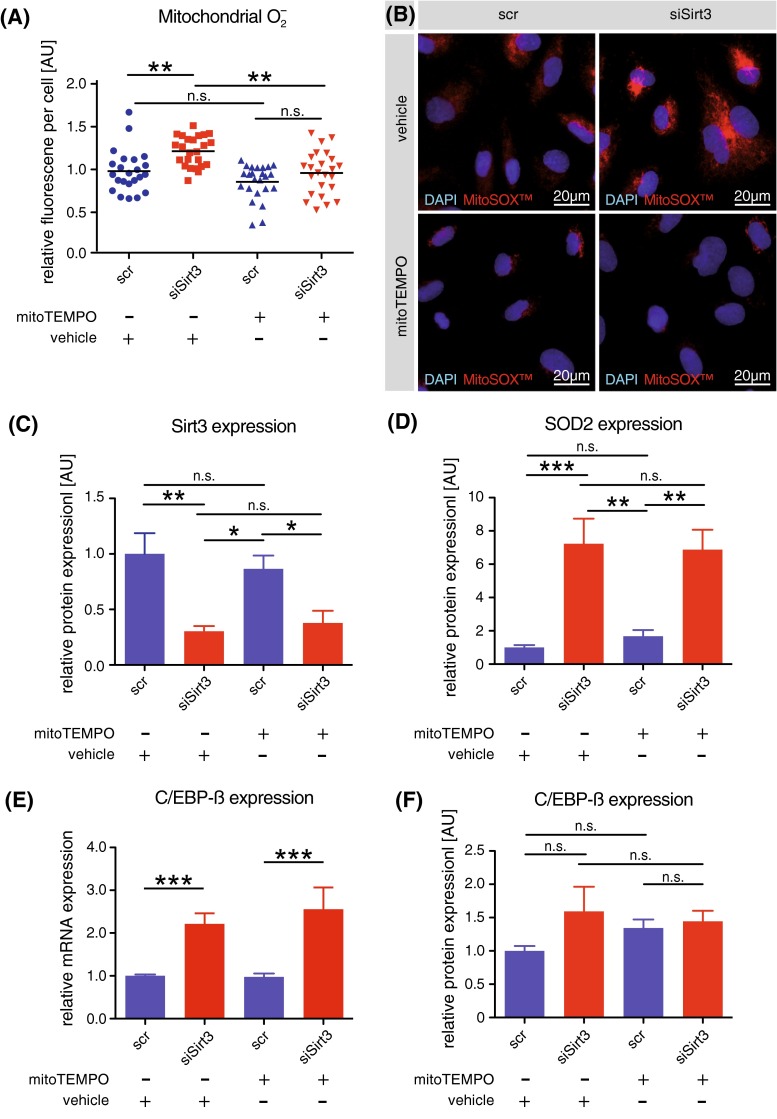
Scavenging mitochondrial superoxide does not affect Sirt3-dependent transcriptional induction of SOD2. **a** Quantification of mitochondrial superoxide per cell, as visualized by MitoSOX™ staining, using fluorescence imaging of HAEC following transient knockdown of Sirt3 or transfection with scrambled siRNA (scr) in presence or absence of the mitochondrial-targeted anti-oxidant mitoTEMPO. **b** Representative micrographs of the setup described in **a**, showing nuclei (*blue*, DAPI) and mitochondrial superoxide (*red*, MitoSOX™). **c**, **d**, **f** Quantification of western blot analyses of Sirt3 (**c**), SOD2 (**d**), and C/EBP-β (**f**). **e** quantitative PCR of C/EBP-β. At least three independent experiments in biological triplicates were performed. *Scale bars* 20 μm, *DAPI* 4′-6-diamidin-2-phenylindol, *n.s.* non-significant, **p < 0.01, ***p < 0.001

To investigate whether SOD2 induction upon Sirt3 deficiency is superoxide-dependent, mitochondrial superoxide was scavenged using the mitochondrial-targeted superoxide scavenger mitoTEMPO. Mitochondrial superoxide accumulation following knockdown of Sirt3 in HAEC was successfully blunted by mitoTEMPO, as assessed by fluorescence imaging after MitoSOX staining (Fig. 5a–c). Interestingly, SOD2 induction upon Sirt3 knockdown was unaffected by blunting mitochondrial superoxide accumulation (Fig. 5d). Transcriptional upregulation of C/EBP-β following knockdown of Sirt3 was also unaltered upon scavenging of mitochondrial superoxide (Fig. 5e). Translation to increased protein levels could not be observed, independent of mitochondrial superoxide (Fig. 5f).

### Interruption of the physiological C/EBP-β-dependent feedback regulation of endothelial SOD2 exacerbates mitochondrial superoxide levels and culminates in endothelial cell death

**Fig. 6:**
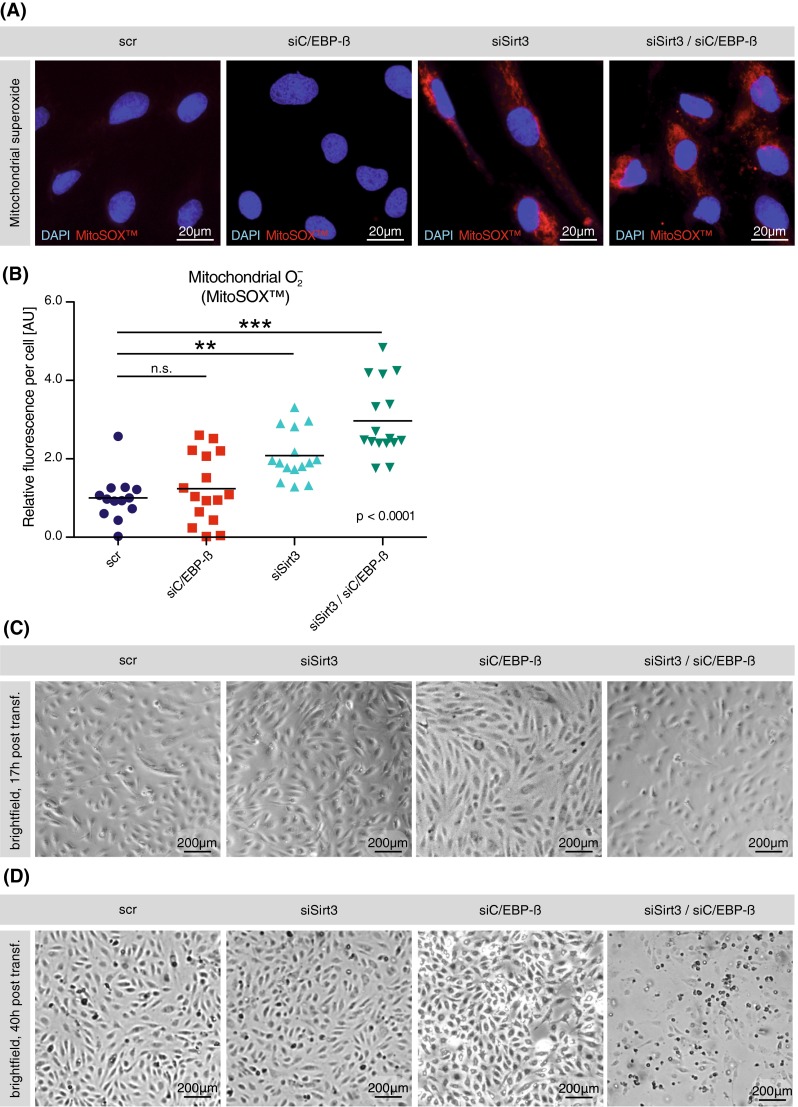
Interruption of the physiological C/EBP-β-dependent transcriptional feedback regulation of SOD2 during transient knockdown of Sirt3 exacerbates mitochondrial superoxide formation and culminates in endothelial cell death. **a** Fluorescence imaging of HAEC following single and simultaneous transient knockdown of C/EBP-β and Sirt3, respectively. Representative micrographs show nuclei (*blue*) and mitochondrial superoxide (red, MitoSOX™), the latter visualized by the mitochondrial- and superoxide-specific fluorescent MitoSOX™ probe. *Scale bars* 20 μm. **b** Quantification of mitochondrial superoxide per cell; medians and single data points are shown. **c**, **d** Representative brightfield phase-contrast micrographs of cultured HAEC 17 h (**c**) and 40 h (**d**) after transient knockdown of Sirt3 and C/EBP-β, either alone or in combination; *scale bars* 200 μm. At least three independent experiments in biological triplicates were performed, *scr* scrambled control, *DAPI* 4′-6-diamidin-2-phenylindol, *n.s*. non-significant, *p < 0.05, **p < 0.01, ***p < 0.001

To reveal the functional relevance of the C/EBP-β-dependent transcriptional feedback regulation of endothelial SOD2 upon Sirt3 deficiency, we assessed mitochondrial superoxide levels following single or simultaneous knockdown of C/EBP-β and Sirt3, respectively, compared with sham-transfected controls. Concomitant with the abrogation of the transcriptional induction of SOD2 following simultaneous knockdown of C/EBP-β and Sirt3, mitochondrial superoxide levels were further enhanced compared with single-knockdown controls (Fig. 6a, b). Transient knockdown of C/EBP-β alone had no effect on mitochondrial superoxide levels (Fig. 6a, b). Interestingly, we observed an increased cell death upon prolonged cultivation (40 h) following simultaneous knockdown of C/EBP-β and Sirt3 that occurred in none of the control conditions (Fig. 6c, d): Incubation for up to 40 h following knockdown led to a demise of the majority of cells (Fig. 6d), which we interpret as the consequence of increased oxidative stress.

## Discussion

### Principle findings

We identify Sirt3 as an important player in the homeostasis of endothelial mitochondrial superoxide. Under physiological conditions, endogenous endothelial Sirt3 appears to uphold SOD2 activity by maintaining its deacetylation for effective degradation of mitochondrial superoxide and thereby preserving normal endothelial function. Lack of Sirt3 is associated with a hyperacetylation of SOD2. A concomitant loss of SOD2 activity is, however, compensated for by a C/EBP-β dependent induction of SOD2 expression. The latter occurs independent of mitochondrial superoxide accumulation, yet potentially upon an initial transcriptional repression of SOD2 upon Sirt3 deficiency. Consequently, endothelial function is maintained under normal circumstances. Only upon a high-cholesterol diet, leading to increased oxidative stress, a mild, superoxide-dependent impairment of endothelial function can be unmasked. In vitro experiments in human aortic endothelial cells indicate that this may be due to an accumulation of mitochondrial superoxide secondary to a residual moderate imbalance of mitochondrial superoxide generation and detoxification. Since none of the superoxide scavenging or generating enzymes assessed were altered upon Sirt3 deficiency, we extrapolate that impaired mitochondrial function upon Sirt3 deficiency [38, 54] may increase mitochondrial superoxide formation.

### Added value

Anti-oxidative effects of Sirt3 have been described in a variety of contexts including age-related hearing loss [49], embryogenesis [22], neuronal injury [10], exercise training [27], and cardiac hypertrophy [51]. In the majority of these settings, the protective effects of Sirt3 are mediated by an augmented radical scavenging through SOD2 and/or catalase. It remains controversial whether these effects are brought about by a transcriptional induction of either of these two ROS detoxification systems or by their Sirt3-dependent deacetylation and consecutive activation [8, 42, 51].

In the present study, we report on the role of endogenous Sirt3 in human aortic *endothelial* cells and its functional effects on *endothelium*-*dependent vasodilation* in a mouse model applying a genetic loss-of-function approach. Corroborating previous reports on anti-oxidative effects of Sirt3 [42], transient knockdown of *endothelial* Sirt3 abrogated the superoxide scavenging capacity of SOD2 and increased its acetylation.

In contrast to previous data [51], we observed that transcription of endothelial SOD2 was not reduced but enhanced following transient knockdown of Sirt3, compensating for its decreased enzymatic activity. However, an initial transcriptional repression of SOD2 upon Sirt3 deficiency, preceding the C/EBP-β dependent transcriptional induction of SOD2 cannot be excluded. Even though the drop in endothelial SOD2 activity upon Sirt3 deficiency slightly exceeded its transcriptional increase, we extrapolate that an excess in mitochondrial superoxide generation secondary to a Sirt3-dependent impairment of mitochondrial function [38] may contribute to the disequilibrium between mitochondrial superoxide generation and detoxification. Of note, expression levels of other endothelial ROS and reactive nitrogen species detoxification systems, including catalase and the thioredoxin system, were unaffected by transient knockdown of Sirt3. Therefore, a thioredoxin-mediated transcriptional induction of SOD2, as reported in yeast and primary human lung microvascular endothelial cells [11, 39] appears unlikely. Assessment of endothelial nitric oxide synthase (eNOS) uncovered a decreased phosphorylation of Thr495 as well as an increased coupling following transient knockdown of Sirt3, equivalent to an increased enzymatic activity [43, 52]. Importantly, NO generation remained unchanged, indicating that increased activity remains functionally irrelevant with regard to NO homeostasis. However, increased eNOS coupling may contribute to counteract increased ROS levels upon Sirt3 deficiency.

Moreover, our in vitro experiments identify mitochondria as the compartment exhibiting increased ROS in the absence of endothelial Sirt3, a finding, for which to date only indirect evidence exists [22, 54].

Elegant promoter studies identified that C/EBP-β is necessary to align with an intronic enhancer of SOD2 to facilitate its transcription in response to increased levels of intracellular ROS [6, 21, 30]. The differential regulation of C/EBP-β in response to transient Sirt3 knockdown and its acetylation-dependent binding capacity [6] prompted us to investigate the role of C/EBP-β in the transcriptional regulation of SOD2 in the absence of endothelial Sirt3. Abrogation of SOD2 induction upon simultaneous knockdown of both Sirt3 and C/EBP-β exacerbated mitochondrial superoxide accumulation and culminated in endothelial cell death after prolonged cultivation. Interestingly, we observed a bidirectional feedback regulation between C/EBP-β and SOD2 with an SOD2-dependent transcriptional induction of C/EBP-β and vice versa. This might indicate that an initial transcriptional repression of SOD2 upon Sirt3-deficiency, as has been reported by others [20, 24, 45, 51], in addition to a blunted SOD2 activity, may have preceded C/EBP-β dependent transcriptional induction of SOD2. Importantly, transcriptional induction of C/EBP-β was independent of mitochondrial superoxide. These findings highlight the functional relevance of this novel C/EBP-β-dependent transcriptional induction of SOD2 in absence of Sirt3 in human aortic endothelial cells.

The ex vivo assessment of endothelium-dependent vessel relaxation showed a rather atypically shaped relaxation curve; the initial sigmoidal shape is interrupted by a weak contraction, starting at acetylcholine levels around 1 µM, before reaching complete relaxation at 100 µM. Interestingly, this intermittent contraction was exacerbated in mice fed a high-cholesterol diet. This and the fact that this phenomenon disappeared in presence of PEG-SOD indicate that this intermittent contraction in response to higher acetylcholine dosages is an oxidative stress-dependent phenomenon, as we have observed previously [60].

### Potential limitations

This study has to be interpreted in light of the following limitations: The difference in endothelium-dependent vasorelaxation between the two genotypes is mild. However, rescue of this phenotype by scavenging of superoxide via exogenous pegylated superoxide dismutase proves superoxide-dependency. Furthermore, improvement of the sensitivity to ACh of both genotypes upon enhanced superoxide scavenging highlights the tight regulation of endothelial function by superoxide and NO and stresses the physiological relevance of Sirt3 and C/EBP-β in regulation of endothelial SOD2 activity and thus of endothelial function.

In light of the C/EBP-β dependent transcriptional compensation of the Sirt3-dependent loss of SOD2 activity in concert with unchanged expression levels of other superoxide generators or scavengers, our data do not provide an explanation for the increased mitochondrial superoxide levels upon Sirt3 deficiency. The extrapolation that a known Sirt3-dependent impairment of the mitochondrial function may enhance mitochondrial superoxide generation remains speculative and warrants further investigation.

In addition, we would like to point out that endothelial dysfunction in Sirt3^−/−^ mice was observed only upon exposure to a high-cholesterol diet, known to induce oxidative stress [37]. In vitro, mitochondrial superoxide accumulation and differential SOD2 regulation upon Sirt3 deficiency was apparent under basal conditions. Though we assume that this is due to the nature of in vitro setups in general, extrapolation to our ex vivo data may be limited.

Moreover, endothelial-dependent vasodilation was assessed using mouse aortic rings in organ chamber baths, which is an ex vivo approach. Again, extrapolation to in vivo vascular function as well as to other species has to be done with caution.

## Conclusions and implications

The current data indicate a protective role of endogenous endothelial Sirt3 in mice fed a high-cholesterol diet, maintaining endothelium-dependent vasorelaxation. The in vitro findings suggest that a novel C/EBP-β-dependent rescue mechanism diminishes *Sirt3*-dependent endothelial dysfunction under physiological conditions (normal diet).

We have reported Sirt3-mediated protection from accelerated weight gain and a decline in metabolic flexibility [55], two important risk factors of human cardiovascular diseases [5]. Our current findings identify an interplay of Sirt3, SOD2, and C/EBP-β in the endothelial redox system. Endothelial dysfunction is independently associated with future adverse cardiovascular events [16, 17, 36, 40, 48]. Further research is warranted to better understand the putatively protective role of Sirt3 in human cardiovascular disease.

## Electronic supplementary material

Below is the link to the electronic supplementary material.

Supplementary material 1 (DOCX 1806 kb)
