# Supplementary material for: Mild endothelial dysfunction in Sirt3 knockout mice fed a high-cholesterol diet: protective role of a novel C/EBP-β-dependent feedback regulation of SOD2
Source: Basic Res Cardiol. 2016 Apr 12;111:33. doi: 10.1007/s00395-016-0552-7 (PMC4829622; doi:10.1007/s00395-016-0552-7)
Supplement: Supplementary file 1 — Supplementary material 1 (DOCX 1806 kb) [file 395_2016_552_MOESM1_ESM.docx]

**Mild endothelial dysfunction in Sirt3 knockout mice fed a high-cholesterol diet**

*Protective role of a novel C/EBP-ß-dependent feedback regulation of SOD2*

Stephan Winnik^1, 2^ *^#^, Daniel S. Gaul^2, 5^ *, Giovanni Siciliani^2^, Christine Lohmann^2^, Lisa Pasterk^2^, Natacha Calatayud^2^, Julien Weber^2^, Urs Eriksson^2, 3^, Johan Auwerx^4^, Lambertus J. van Tits^2^, Thomas F. Lüscher^1, 2, 5^, Christian M. Matter^1, 2, 5 #^

* equal contribution

^1^ University Heart Center Zurich, Department of Cardiology, University Hospital Zurich, Zurich, Switzerland

^2^ Center for Molecular Cardiology, University of Zurich, Schlieren, Switzerland

^3^ Division of Cardiology and Department of Medicine, GZO Regional Health Center Wetzikon, Wetzikon, Switzerland

^4^ Laboratory of Integrative Systems Physiology, School of Life Science, Ecole Polytechnique Fédérale de Lausanne, Lausanne, Switzerland

^5^ Zurich Center for Integrative Human Physiology, University of Zurich, Zurich, Switzerland

**Supplementary Material / Online Resource**

**Running title:** *Sirt3 in Endothelial Function*

Key words: *Sirt3, oxidative stress, SOD2, C/EBP-ß, endothelial function*

^#^ Correspondence:

| Stephan Winnik, M.D., Ph.D.  University Heart Center Zurich  Department of Cardiology  University Hospital Zurich  Raemistr. 100, 8091 Zurich  Switzerland  E-mail: stephan.winnik@usz.ch | Christian M. Matter, M.D.  University Heart Center Zurich  Department of Cardiology  University Hospital Zurich  Raemistr. 100, 8091 Zurich  Switzerland  E-mail: christian.matter@usz.ch |
| --- | --- |

**Figure S1** – *Aortic relaxation is endothelium- and nitric oxide (NO)-dependent*

**Figure S1** – Relaxation of aortic rings from *wild-type* and *Sirt3^-/-^* mice in response to sodium nitroprusside (SNP) following a normal diet (A) or a high cholesterol diet (B). (C) Relaxation of aortic rings from *wild-type* and *Sirt3^-/-^* mice fed a normal diet in response to acetylcholine (ACh) after preincubation with L-nitroarginine methyl ester (L-NAME), an inhibitor of endothelial nitric oxide synthase. n = 9 to 11 per group, quantification of the areas under the curve (AUC), boxplots show interquartile ranges, whiskers indicate minima and maxima.

**Figure S2** – *Body weight does not differ between Sirt3^-/-^* and *wildtype* controls

**Figure S2** – (A) Body weights of *wild-type* and *Sirt3^-/-^* mice before subjecting them to organ chamber experiments.

**Figure S3** – *Expression of glutathione peroxidase, xanthine oxidase, thioredoxin 1 and 2, and thioredoxin-dependent peroxide reductase are unaltered following transient knockdown of Sirt3*

**Figure S3** – Expression analyses of (A) glutathione peroxidase, (B) xanthine oxidase, (C) thioredoxin 1, (D) thioredoxin 2, (E) thioredoxin-dependent peroxide reductase (PRDX3), and (F, G) NADPH oxidase subunits p47*^phox^* and p22*^phox^* in HAEC following transient knockdown of Sirt3, using quantitative PCR (A, C-G left panel) and western blot analysis (B, G right panel), respectively. At least three independent experiments in biological triplicates were performed. Scr = scrambled control.

**Figure S4** – *Nitric oxide (NO) generation is not affected by Sirt3 deficiency*

**Figure S4** – Expression analyses (western blot) of (A) total eNOS, (B) eNOS phosphorylated at serine 1177 (p-eNOS(Ser1177)), and (C) eNOS phosphorylated at threonine 495 (p-eNOS(Thr495)) in HAEC upon knockdown of Sirt3 or control transfection using scrambled siRNA (scr). (D) Western blot analyses of eNOS un-/coupling upon knockdown of Sirt3 or control transfection using scrambled siRNA (scr). (E) Nitric oxide production using DAF-2 diacetate in HAEC upon knockdown of Sirt3, control transfection using scrambled siRNA (scr) or non-transfected (NT) controls. Each condition was assessed with or without (vehicle) L-NIO, a non-selective nitric oxide synthase inhibitor. ***) p<0.001, **) p<0.01, n.s. = not significant. Boxpots show interquartile ranges, whiskers indicate minima and maxima.

**Figure S5** – *Loss of SOD2 induces transcription of C/EBP-ß*

**Figure S5** – Expression analyses using quantitative PCR (left hand side) and western blot analysis (right hand side) of SOD2 (A, B), C/EBP-ß (C, D), and Sirt3 (F, G) of HAEC following transient knockdown of SOD2 (siSOD2) or control transfection with scrambled siRNA (scr). At least three independent experiments in biological triplicates were performed.
